# Chemotherapy and Quality of Life: An Observational Study of Cancer Patients at the Oncology Unit of Tertiary Care Hospital in Islamabad

**DOI:** 10.1055/s-0045-1814102

**Published:** 2025-12-29

**Authors:** Sameen Abbas, Kanza Zahid, Bilal Ashraf, Muhammad Asim Ali, Muhammad Nouman Asghar, Amna Shahid, Kainat Hameed

**Affiliations:** 1 Department of Pharmacy, Quaid-i-Azam University, Islamabad, Pakistan; 2 Department of Medicine, Rawalpindi Medical University, Rawalpindi, Pakistan; 3 Faculty of Pharmacy, Bahauddin Zakariya University, Multan, Pakistan; 4 Faculty of Pharmacy, Gomal University, Dera Ismail Khan, Pakistan

**Keywords:** cancer, chemotherapy, quality of life, oncology, Pakistan

## Abstract

**Background:**

Cancer is a global health challenge, with millions of new cases annually. While improving survival rates, the disease and its treatment, particularly chemotherapy, significantly impact patients' quality of life (QoL) and cause side effects that severely affect patients' physical, emotional, and social well-being. As the number of cancer patients is rising in Pakistan, the health care system still faces multiple issues that may have an impact on patient outcomes and QoL. So, this study aims to assess the QoL of cancer patients before and after chemotherapy at a tertiary care hospital in Islamabad.

**Methodology:**

A prospective cohort study at PIMS, Islamabad, enrolled 184 chemotherapy patients using consecutive sampling techniques. Ethical approvals and informed consent were obtained. Clinical as well as demographic data, together with well-being (Functional Assessment of Cancer Therapy–General [FACT-G]), were analyzed using SPSS version 21.

**Results:**

Of 184 patients, 71.9% had hematological malignancies and 28.1% solid tumors. FACT-G scores demonstrated significant domain-specific improvements following chemotherapy, in which physical and functional well-being improved significantly ( *p* = 0.008 and 0.01, respectively), while emotional and social well-being showed modest but nonsignificant changes. Additionally, marked improvements were noted in hematological, renal, and hepatic parameters that directly influence the health-related QoL.

**Conclusion:**

Chemotherapy significantly improved QoL in specific domains, particularly physical and functional well-being, though emotional and social challenges persisted. These findings highlight the need for early detection, comprehensive supportive care, and survivorship programs tailored to the Pakistani context in mitigating these impacts and enhancing patient well-being.

## Introduction

Cancer is a noteworthy global health challenge, with new cases diagnosed in millions each year. Among the various treatment modalities, chemotherapy remains one of the most common, even though it has a spectrum of side effects that can severely affect patients' physical, emotional, and social well-being. 1 It is essential to understand these effects to maximize care and support for cancer patients. Although survival rates have increased, the illness and its treatment still have a significant negative impact on patients' quality of life (QoL). 2 Chemotherapy, while targeting cancer cells, often causes adverse effects such as fatigue, nausea, pain, and emotional distress, which can collectively diminish patients' QoL. 3

QoL in cancer patients is a multifaceted concept, encompassing various dimensions such as physical functioning, social interactions, psychological state, and overall well-being. Studies have shown that interventions aimed at alleviating chemotherapy-induced side effects can significantly enhance QoL, underlining the need for routine QoL evaluations in clinical practice. 3 However, there is a lack of data specific to the Pakistani context, where social, cultural, and economic factors may exclusively influence patients' experiences and perceptions of QoL.

In Pakistan, the burden of cancer is rising, with increasing numbers of patients requiring chemotherapy. 4 Most often, two or more chemotherapeutic drugs are administered, also known as combination chemotherapy, and form the basis of most chemotherapy today. The rationale for using combination chemotherapy is that the different drugs enhance each other's effects and create a better synergistic effect than if they were used as single agents. 5 According to a study, chemotherapy can be used as a single treatment modality but is also commonly used in combination with surgery, radiotherapy, and biological treatment. 6 Some of the widely approved protocol regimens observed are mentioned in Table 1 . Despite this, there is limited evidence comparing the QoL of patients receiving protocol versus nonprotocol chemotherapy regimens.

**Table 1:** List of different chemotherapeutic protocol regimens

Name	Components	Uses
DA or DAC	7 days of cytarabine plus 3 days of daunorubicin	Acute myelogenous leukaemia
ABVD	Doxorubicin (Adriamycin), bleomycin, vinblastine, dacarbazine	Hodgkin's lymphoma
CHOP	Cyclophosphamide, doxorubicin, vincristine (Oncovin), prednisone	Non-Hodgkin lymphoma
CMF	Cyclophosphamide, methotrexate, 5-FU	Breast cancer
MOPP	Mechlorethamine, vincristine (Oncovin), procarbazine, prednisone	Hodgkin's lymphoma
FOLFOX	Fluorouracil (5-FU), folinic acid (Leucovorin), oxaliplatin	Colorectal cancer
CA	Cyclophosphamide, doxorubicin (Adriamycin)	Breast cancer

In modern oncology, assessing the effectiveness of chemotherapy involves not only life expectancy measures, such as cancer-specific and overall survival, but also the patient's subjective evaluations of well-being after treatment. Even with advancements, Pakistan's health care system still faces issues such as inadequate resources, inconsistent access to care, and socioeconomic differences that may have an impact on patient outcomes and QoL. Health-related quality of life (HRQoL) is a crucial therapeutic goal for cancer patients and their providers. The Functional Assessment of Cancer Therapy–General (FACT-G) is a widely used tool for assessing HRQoL in cancer patients. 7 Developed and validated years ago, the FACT-G has been translated into nearly 50 languages and comprises four subscales: physical well-being, social/family well-being, emotional well-being, and functional well-being. 8 For this purpose, it was aimed to assess QoL among cancer patients before and after chemotherapy at a tertiary care hospital in Islamabad, with particular focus on domain-specific changes between protocol-based and nonprotocol regimens.

## Methodology

A prospective cohort study was performed from May 1, 2023, to November 25, 2024, at the Department of Oncology, PIMS, Islamabad. A total of 184 patients were registered using a consecutive/total enumerative sampling strategy based on the inclusion criteria. Newly diagnosed patients with cancer, both male and female, above the age of 18, receiving chemotherapy, were included. However, patients who were unreachable or unwilling to respond and had not given consent were omitted. Additionally, patients receiving treatment other than chemotherapy, such as radiotherapy or any surgical procedure, were excluded.

### Ethical Consideration

This study was approved by the Bio-Ethical Committee (BEC) of Quaid-i-Azam University and the Institutional Review Board committee of PIMS Hospital in written form. Informed written consent was obtained from each patient, and they were informed that this information would be used only for academic research purposes and that no harm or risks would be incurred.

### Data Collection Tool

A questionnaire was used to collect data, covering demographics such as age, gender, weight, height, marital status, location, education, and income level of the cancer patients. The second part included well-being parameters for HRQoL (physical, social, emotional, and functional) from the prevalidated FACT-G questionnaire, scored on a Likert scale. Individual well-being parameters were scored from 0 to 28, with total FACT-G scores ranging from 0 to 108. 7 8 9

### Data Collection

The data collection process involved questionnaire filling by patients and self-analysis of patient charts. Two visits were conducted for each participant: one at the start of treatment and another after the end of each chemotherapeutic session, irrespective of doses. Data collection took place in the OPD during regular check-ups and consultations, as well as in the chemotherapy room.

### Data Handling

All the data collected from the participants were clarified and checked to ensure that all the necessary information for the study had been properly collected and recorded. The completed questionnaire was coded for analysis and entered into MS Excel. The scores were 0 to 12 = low, 13+ = high for individual well-being parameters, 0 to 54 = low, and 55+ = high for total FACT-G scores. Scoring was done according to defined rules. 8 9 10 All the data were collected individually at pre- and postchemotherapeutic levels.

### Statistical Analysis

The data were exported to SPSS (IBM version 21) to describe the data and identify the study's results. Descriptive statistics and a graphical summary of demographic information, such as sex, age, education, marital status, and socioeconomic status, in the form of charts (pie charts, bar charts) were done. Wilcoxon's signed-rank test and paired sample *t* -test were used for the analysis of QoL before and after chemotherapy. The level of statistical significance was *p* < 0.05.

## Results

Out of the total 184 sample population, 71.9% of the population had blood cancers, and the remaining 28.1% were suffering from solid cancers, with 71.9% of the population having blood cancers, including 27% acute lymphoblastic leukemia (ALL) and 18.7% acute myeloid leukemia (AML), with small percentages of chronic lymphocytic leukemia, chronic myelogenous leukemia, non-Hodgkin lymphoma, and multiple myeloma. As 28.1% were suffering from solid cancers, including 11.5% breast cancer, 6.3% ovarian cancer, 3.1% gastric cancer, and the remaining minor values come under lung and liver cancer patients, as summarized in Table 2 .

**Table 2:** Different cancer types in the study population

Type of cancers	Frequency	Percentage
ALL	50	27
AML	34	18.7
Breast	21	11.5
NHL	17	9.2
MM	11	7.3
CLL	10	6.3
Ovarian	10	6.3
HL	10	6.3
Lung	7	4.2
Gastric	6	3.1
Liver	4	2.1
CML	2	1
HCL	2	1
Total	184	100

Abbreviations: ALL, acute lymphocytic leukemia; AML, acute myelogenous leukemia; CLL, chronic lymphocytic leukemia; CML, chronic myelogenous leukemia; HCL, hairy cell leukemia; HL, Hodgkin's lymphoma; MM, multiple myeloma; NHL, non-Hodgkin's lymphoma.

### Treatment Plan

A total of 85.4% of patients received chemotherapy alone, while 9.4% received radiotherapy, 4.2% underwent surgical procedures, and only 1% received a combination of radiation, surgery, and chemotherapy. Out of these 184 study respondents, patients receiving regimens according to protocols constitute only 31.3%, while nonprotocol regimens constitute 68.8%.

### HRQOL before and after Treatment

HRQoL was observed before and after chemotherapy was given to the patient. The Wilcoxon's signed-rank test was applied to find any significant impact of chemotherapy on the patient's HRQoL, as shown in Table 3 . It was observed that physical, functional, and overall well-being improved significantly after the chemotherapeutic sessions.

**Table 3:** Wilcoxon's signed-rank test at pre- and postdose levels for HRQoL domains

	Prechemotherapy mean (SD) *N* = 184		Postchemotherapy mean (SD) *N* = 184		*p* -Value
Physical well-being	15.95 (4.24)	H	18.11 (1.96)	H	0.008 a
Social well-being	12.78 (3.04)	L	13.90 (2.76)	H	0.305
Emotional well-being	15.96 (3.62)	H	16.05 (2.62)	H	0.554
Functional well-being	9.64 (3.87)	L	15.94 (3.77)	H	0.01 a
Overall sum	54.33 (9.85)	L	69.94 (7.8)	H	0.04 b

Abbreviations: H, high; HRQoL, health-related quality of life; L, low; SD, standard deviation.

a Significant *p* ≤ 0.01.

b Significant *p* ≤ 0.05.

### Investigation of HRQOL of Chemotherapeutics Given According to Protocols Compared with That of Nonprotocol One

The study found significant improvements in the HRQoL of cancer patients undergoing specific chemotherapy regimens, including increased physical and functional well-being in patients with ALL and AML who received these regimens. Additionally, breast cancer patients undergoing specific chemotherapy regimens also experienced changes in functional well-being. The key findings of our study are summarized in Table 4 . It was noted that cancer patients undergoing specific chemotherapy regimens, including those with ALL, AML, and breast cancer, experienced improved HRQoL.

**Table 4:** Pre- and post-D results of different chemotherapeutic regimens for HRQoL domains

Cancer type	Regimens	HRQoL domains																			
		PWB				SWB				EWB				FWB				Overall sum			
		Pre-D M (SD)		Post-D M (SD)		Pre-D M (SD)		Post-D M (SD)		Pre-D M (SD)		Post-D M(SD)		Pre-D M (SD)		Post-D M (SD)		Pre-D M (SD)		Post-D M (SD)	
ALL ( *N* = 50)	Larson ( *N* = 23)	14.09 (4.92)	H	17.83 (1.12) a	H	11.48 (3.16)	L	12.91 (3.09)	L	15.00 (3.81)	H	16.47 (3.37)	H	8.58 (2.94)	L	16.17 (3.4) b	H	61.17 (9.89)	H	70.09 (6.87) a	H
	Larson excluding L-asparaginase ( *N* = 10)	14.27 (3.70)	H	15.61 (1.21)	H	12.26 (2.04)	L	14.70 (2.17)	L	13.97 (3.14)	H	15.74 (2.25)	H	12.85 (1.21)	L	15.71 (2.94) a	H	56.71 (8.98)	H	60.78 (8.77)	H
	Nonprotocol ( *N* = 17)	17.68 (4.29)	H	12.88 (2.32) a	L	10.87 (3.16)	L	11.91 (2.38)	L	18.9 (3.84)	H	16.47 (3.37)	H	13.65 (2.94)	H	16.17 (3.04)	H	71.16 (9.89)	H	70.09 (6.87)	H
AML ( *N* = 34)	7 + 3 Regimen ( *N* = 23)	12.27 (4.22)	L	18.79 (2.36) b	H	12.08 (3.41)	L	15.86 (3.35)	H	16.21 (4.41)	H	16.57 (2.44)	H	11.14 (3.55)	L	16.43 (4.70) a	H	65.71 (10.3)	H	69.79 (9.99)	H
	Nonprotocol ( *N* = 11)	14.27 (3.70)	H	15.61 (1.21)	H	13.26 (2.04)	H	13.70 (2.17)	H	13.97 (3.14)	H	15.74 (2.25)	H	12.85 (1.21)	L	15.71 (2.94)	H	56.71 (8.98)	H	60.78 (8.77)	H
Breast cancer ( *N* = 21)	CA ( *N* = 10)	16.50 (2.63)	H	19.20 (1.39) a	H	10.25 (2.5)	L	12.10 (2.6) a	L	15.44 (2.96)	H	16.56 (2.35)	H	13.50 (4.33)	H	20.10 (2.77) a	H	66.35 (9.39)	H	73.67 (7.27)	H
	CMF ( *N* = 11)	15.33 (3.33)	H	12.00 (0.63) b	L	8.86 (4.20)	L	10.00 (4.38)	L	14.83 (3.19)	H	16.58 (3.87)	H	16.67 (3.09)	H	11.00 (3.2) b	L	63.03 (9.72)	H	60.50 (6.56)	H
NHL ( *N* = 17)	CHOP ( *N* = 10)	15.50 (5.67)	H	16.25 (1.93)	H	13.04 (2.40)	H	13.00 (2.45)	H	14.25 (4.86)	H	15.50 (1.00)	H	8.75 (4.65)	L	16.50 (4.12) a	H	61.54 (15.5)	H	71.08 (9.08) a	H
	CHOP excluding oncovin ( *N* = 7)	16.00 (6.98)	H	18.75 (1.50)	H	11.87 (4.40)	L	12.50 (4.20)	L	15.50 (4.36)	H	15.00 (1.00)	H	9.25 (4.35)	L	12.25 (3.86)	H	62.65 (15.8)	H	68.17 (9.89)	H

Abbreviations: ALL, acute lymphocytic leukemia; AML, acute myelogenous leukemia; CA, cyclophosphamide, doxorubicin; CHOP, cyclophosphamide, doxorubicin, vincristine, prednisone; CMF, cyclophosphamide, methotrexate, fluorouracil; D, dose; EWB, emotional well-being; FWB, functional well-being; H, high; HRQoL, health-related quality of life; L, low; M, mean; NHL, non-Hodgkin lymphoma; PWB, physical well-being; SD, standard deviation; SWB, social well-being.

a Significant *p* ≤ 0.05.

b Significant *p* ≤ 0.01.

### Signs and Symptoms of Cancer

Some patients came with complaints of different signs they were experiencing due to the cancer they were suffering from. When chemotherapeutic agents were administered, many patients experienced various side effects, including complaints, signs, or symptoms, depending on the drug, dose, and patient's physical condition. Many cancer patients were experiencing more than one of the below-mentioned signs and symptoms, which together led to more complications even before treatment, as shown in Table 5 .

**Table 5:** Signs and symptoms by sample population

	Signs and symptoms ( *N* = 184)	Postdose signs and symptoms ( *N* = 184)
Muscle pain	106 (57.6)	93 (50.5) a
Fatigue	99 (53.8)	72 (39.1) a
Nausea and vomiting	96 (52.2)	84 (45.6) a
Joint pain	94 (51.1)	52 (28.3) a
Cough	74 (40.2)	50 (27.2) a
Mouth sores	68 (37.0)	40 (21.7) a
Breathlessness and looking pale	52 (28.3)	49 (24.4)
Urinary complications	28 (9.8)	15 (8.1) a
Loss of appetite	40 (21.8)	29 (15.8) a
Diarrhea	36 (19.6)	26 (14.1) a
Tingling numbness	26 (14.1)	24 (13.04)
Presence of dry eyes and dry mouth	26 (14.1)	19 (10.3)
Alopecia	14 (7.6)	14 (7.6)
Bruising, bleeding gums, or nosebleeds	14 (7.6)	10 (5.4)
Skin rash	4 (2.2)	1 (0.54)

a Significant *p* ≤ 0.05.

### Laboratory Parameters before and after Chemotherapy

Table 6 shows the results of cancer patients receiving chemotherapy who underwent blood examinations for hematological parameters, renal and liver function tests, and electrolyte levels. When the impact of chemotherapy was observed on these parameters, significant effects were observed on each of these parameters. Individually observed from a paired *t* -test, red blood cell and platelet counts with hemoglobin were significantly decreased from normal to low values, whereas white blood cell count came under normal from below-normal range. Creatinine values increased significantly with sodium and potassium salt values, but no effect was seen on calcium salt levels due to chemotherapy. Additionally, the total bilirubin level of cancer patients was not affected by the overall chemotherapy administered to them, as statistically proven. Alanine transaminase and aspartate transaminase levels increased after chemotherapy, but albumin levels decreased.

**Table 6:** Pre- and postdose results of chemotherapy for different laboratory parameters

		Normal values	Prechemotherapy, mean (SD)		Postchemotherapy, mean (SD)		*p* -Value
Hematological parameters	RBC count (million/µL)	3.5–5.6 million/µL	4.86 (4.24)	Normal	3.28 (1.96)	Low	0.02 a
	WBC count (/µL)	4–11 × 10 ^3^ /µL	5.2 (1.9)	Normal	10.3 (2.5)	Normal	0.005 b
	Platelet count (/µL)	1.4–4 × 10 ^5^ /µL	1.8 (1.3)	Normal	1.0 (1.4)	Low	0.034 a
	Hemoglobin (g/dL)	11–18 g/dL	12.95 (1.69)	Normal	8.85(1.62)	Low	0.047 a
Renal function test	Calcium (mg/dL)	8.4–10.2 mg/dL	8.7 (1.19)	Normal	8.70 (1.28)	Normal	0.240
	Sodium (mEq/L)	136–146 mEq/L	138.26 (13.05)	Normal	169.5 (13.27)	High	0.036 a
	Potassium (mEq/L)	3.5–5.1 mEq/L	4.59 (0.42)	Normal	4.65 (0.43)	High	0.007 b
	Creatinine (mg/dL)	0.5–1.1 mg/dL	2.2s1 (1.1)	High	2.65 (1.01)	High	0.009 b
Liver function test	Bilirubin (mg/dL)	0.3–1.2 mg/dL	1.11 (0.31)	Normal	0.98 (0.32)	Normal	0.077
	ALT (U/L)	7–55 U/L	29.1 (5.0)	Normal	32.3 (9.1)	Normal	0.09
	AST (U/L)	8–48 U/L	31.6 (8.2)	Normal	42.8 (9.89)	Normal	0.01 a
	Albumin (g/dL)	3.5–5.0 g/dL	3.7 (1.2)	Normal	2.8 (0.9)	Low	0.04 a

Abbreviations: ALT, alanine transaminase; AST, aspartate transaminase; RBC, red blood cell; WBC, white blood cell.

a Significant *p* ≤ 0.05.

b Significant *p* ≤ 0.01.

## Discussion

This research investigates the effects of chemotherapy on QoL following chemotherapy. Traditionally, the effectiveness of chemotherapy has been evaluated based on tumor response, toxicity, and survival rates, with QoL being challenging to measure. 11 Chemotherapy, while essential for treating various cancers, often results in significant physical, emotional, and social challenges for patients. Today, advanced tools quantify QoL, encompassing physical, psychological, and social well-being, which is crucial for informed treatment decisions. 12 13

As observed in our results, patients undergoing chemotherapy reported substantial declines in physical well-being, consistent with previous studies. Common side effects such as fatigue, nausea, and pain were prevalent, significantly impairing daily activities and overall physical health. This aligns with research by Oh and Cho (2020), which found that chemotherapy-related fatigue is a predominant factor in reducing QoL in cancer patients. 14 Notable deficiencies in emotional and social support in the form of anxiety, depression, and emotional distress were observed in our study. Similar conclusions were reported by another study, connecting depression and lack of support to poorer QoL in younger cancer patients. 15 Our results support the findings of various studies, which observed that cancer patients undergoing chemotherapy frequently report diminished emotional and social well-being. 16

QoL is now a critical outcome in clinical trials, supplementing conventional measures such as survival and morbidity. 8 17 Over 80% of our patients reported experiencing fatigue before treatment. The advanced disease often leads to unfavorable outcomes, with malnutrition and cachexia common in late-stage cancer. 18 Early-stage cancer treatments tend to be less complex and less costly, thereby reducing the overall impact on patients' QoL. Furthermore, symptom management and improving QoL depend heavily on the function of comprehensive care, which includes palliative and supportive care. As part of a comprehensive approach to cancer treatment, the World Health Organization emphasizes the need for access to palliative care and pain relief.

Additionally, the study found that patients who received no treatment or were in a diseased state experienced a lower QoL, with minimal impact from oncological treatments or supportive care.

Another key observation from this study is the difference in outcomes between protocol-adherent and nonprotocol chemotherapy regimens. Patients treated under standardized protocols demonstrated more consistent improvements in QoL domains compared with those receiving nonprotocol regimens. Protocol adherence ensures evidence-based dosing schedules, timely monitoring, and integration of supportive care, which likely reduces treatment-related toxicity and improves tolerance. In contrast, nonprotocol regimens may be associated with greater variability in side effects, suboptimal supportive measures, and inconsistent monitoring, resulting in less predictable QoL outcomes. These findings underscore the importance of maintaining adherence to established treatment protocols, particularly in resource-limited settings, to optimize patient outcomes.

This finding suggests that while chemotherapy has significant side effects, the absence of treatment leads to an even greater decline in QoL. This is in line with the work of Haslam et al (2020), which indicated that untreated cancer patients often experience severe declines in QoL due to the progression of disease symptoms. 19 Therefore, preserving QoL is crucial throughout cancer treatment, underscoring the need for comprehensive care approaches that address both physical and psychosocial aspects.

## Limitations of the Study

This study has several limitations. Being conducted at a single tertiary care hospital in Islamabad, the findings may not be generalizable to other settings with different patient populations and health care resources. Furthermore, QoL was assessed only before and immediately after chemotherapy, which did not capture long-term changes or survivorship issues. Although consecutive sampling minimized selection bias, the absence of a priori sample size calculation limited the statistical power, particularly for subgroup analyses between protocol and nonprotocol regimens.

## Conclusion

This study demonstrates that chemotherapy leads to domain-specific changes in QoL, particularly in terms of social and physical functioning, while emotional and social domains showed limited or no improvement. With higher survival rates resulting in more extended hospital stays, complications, and costs, cancer is becoming more often recognized as a chronic condition. These findings highlight the need for supportive interventions beyond chemotherapy to address the persistent psychosocial challenges faced by patients. Given the short-term nature of this study, long-term follow-up is necessary to evaluate the sustained effects of chemotherapy on QoL. Comprehensive cancer care in Pakistan should integrate early detection, standardized treatment protocols, and supportive services, including palliative care and survivorship programs, to better address the multifaceted needs of patients.
